# Attitudes towards the public-private mix for retirement income in Europe

**DOI:** 10.1177/09589287251345904

**Published:** 2025-06-03

**Authors:** Tobias Wiß, Juan J Fernández, Karen M Anderson

**Affiliations:** 27266 Johannes Kepler University Linz, Austria; University Carlos III of Madrid, Spain; 8797 University College Dublin, Ireland

**Keywords:** attitudes, comparative welfare state analysis, pension preferences, pensions, public opinion

## Abstract

Pension systems in affluent democracies are undergoing a process of privatisation and marketisation that will likely reinforce multi-pillar pension systems. Yet, most public opinion research so far investigates attitudes concerning parametric aspects of public pension programmes (pension spending, pension age, level of redistribution). We have very little understanding of individual preferences regarding the mix of public and non-public sources of their own retirement income. This study begins to fill this gap through an analysis of the preferred mix of pension income with the research question: What explains public preferences on the public-private pension mix? Using a novel cross-national survey in six European countries (Austria, Denmark, Germany, Ireland, the Netherlands and Spain), we assess the role of self-interest, ideology and institutional factors in shaping retirement income preferences. We find that self-interest (e.g. age), political ideology, and institutional arguments (i.e. national pension system design) explain pension income preferences.

## Introduction

Many, if not most, pension systems in affluent democracies were introduced in favourable economic and demographic contexts. Robust fertility rates and favourable macroeconomic conditions facilitated the adoption and expansion of public pension schemes covering all or most workers and their dependents. As populations age, the capacity of pension systems to continue to deliver pension security has been called into question. Several affluent democracies have responded to these challenges by adopting reforms that increase the share of non-state pension provision (occupational and private pensions) in the overall public-private pension mix (Ebbinghaus and Wiß, 2024).

We define the public-private mix as the combination of pension income sources an individual is entitled to. This can include public pension schemes, occupational pension arrangements, and/or individual private pension savings products. We classify occupational and individual pensions as “private” or “market-based” or “non-state.”

Research on individual attitudes about pension policy has generated important findings about how individuals evaluate the level of public pension spending and the responsibility of the state for providing retirement income (Ebbinghaus and Naumann, 2020; Fernández, 2013; Jaime-Castillo, 2013), the preferred and expected retirement age (Fernández and Jaime-Castillo, 2013; Hofäcker, 2015; Radl and Fernández, 2022), and the level of redistribution in public pension systems (Breunig et al., 2022; Reeskens and van Oorschot, 2013). However, we have comparatively little knowledge about individual preferences regarding the preferred mix of public and private sources of retirement income. First, existing research is marked by a mismatch between predominant configurations of pension provision in Europe and research on pension attitudes. Most European pension systems are multipillar, yet most attitudinal research focuses on preferences regarding only one pillar: the public one. As a result, the literature presents an incomplete understanding of popular views regarding pension provision. Second, this focus provides a necessary foundation for understanding public support for the mix between different pension income sources (pillars). Pension reforms frequently appear on government agendas, and public support is crucial for their success. Preferences for a certain pension mix may influence voting behaviour, shape political debates, and affect the feasibility of policy changes. Improving our understanding regarding the preferred pension mix is critical because it sheds light on whether the current system and planned reforms align with public opinion.

We are aware of only two studies that investigate public opinion about the sources of pension income. Gelissen (2001) and van Groezen et al. (2009) analyse individual preferences for pension provision by the state, employers, and individual arrangements in 11 European countries, and 15 European countries, respectively. For all their merits, both studies focus on preferences concerning only one source of pension income (the state, employer/industrial relations, private plans) and do not investigate individuals’ preferences for the mix of these three types of provision. Moreover, both studies rely on data from the 1990s and 2001, respectively, so we do not know how recent reforms in the countries studied affect preferences.

This paper addresses these weaknesses in the existing literature and contributes to research in two ways. First, we theorise how individuals evaluate the balance between public, occupational, and private pension provision. Second, we rely on new data about individual attitudes about their preferred pension mix – that is the share of retirement income coming from public, occupational, and private pension plans. We estimate linear and multinomial regression models for data generated in an original online survey fielded in 2023 in six European countries (Austria, Denmark, Germany, Ireland, the Netherlands, and Spain) that vary in their public-private pension mix.

The next section reviews existing research on attitudes towards pension policy, highlighting key determinants and gaps in the literature. We develop our hypotheses in line with previous findings for self-interest, political ideology, feedback effects and institutional context. The paper then describes our data and analytical techniques used to test the hypotheses. We afterwards present the empirical findings, examining how our theoretically informed determinants influence individual attitudes towards the retirement income share of public, occupational, and individual pensions. The last section interprets the key findings in the context of the existing literature and discusses policy implications.

## Individual pension preferences

A growing literature analyses individual social policy preferences across a range of dimensions like demand for redistribution, preferences for higher or lower public social spending and aspects of benefit structure like the conditions of benefit access. This research (e.g., Busemeyer, 2024; Jaime-Castillo, 2013; Lynch and Myrskylä, 2009) identifies three broad sets of determinants: material self-interest, ideology, and institutional context. We draw on this research to try to understand the drivers of individual preferences regarding the public-private mix in their own expected pension income. Although the bulk of this research focuses on individual preferences regarding public social provision, as we note below, there are good reasons to assume that the drivers of public pension policy preferences are highly relevant for how individuals think about their preferred mix of pension income sources.

### Self interest

A vibrant strand of research argues that social policy preferences are driven by individual cost-benefit calculations. Based on the Meltzer-Richard model (Meltzer and Richard, 1981), individuals tend to support policies that provide them with disproportionate benefits and oppose those from which they have disproportionate contributions. Moreover, the degree and type of risk exposure, for example towards financial and labour market risks, determine social policy attitudes (Iversen and Soskice, 2001; Rehm, 2009). Thus, individuals are more likely to favour policies that enhance their own well-being and financial security while resisting policies perceived as irrelevant or disadvantageous to their personal circumstances. To this effect, income, education, age, and gender shape individual interests in the pension mix and can therefore affect public opinion on preferences for the (future) pension income mix.

Research shows that higher income and education levels correlate with a preference for occupational and private pension schemes due to better financial literacy and investment capacity (Boeri et al., 2001; van Groezen et al., 2009). Conversely, low-income individuals tend to favour public pensions for their stability and predictability. High-income individuals are more inclined toward occupational and individual pensions than low-income individuals because public pension schemes often come with a benefit cap (Ebbinghaus and Naumann, 2020; OECD, 2023) providing lower replacement rates to higher than low-income earners (European Commission, 2024) which makes additional savings necessary for a decent old-age living standard. Furthermore, higher incomes enable high-earners to save for retirement through occupational and private pensions. High-income earners also benefit in particular from tax incentives for private pension schemes (Agulnik and Grand, 1998; Sinfield, 2000). Furthermore, individual pensions and some occupational pension plans allow greater control over investment choices and strategies, aligning with the preferences of high-income individuals who either can make more well-informed decisions thanks to higher financial literacy or who can afford financial advice and tailored investment plans.

These findings clash with those of several earlier studies, however. Gelissen (2001) finds little support for the decisive role of income for individuals’ preferred pension income source; Jaime-Castillo (2013) identifies no relationship between education and preferences regarding the extent of private pensions (Jaime-Castillo, 2013); and Lynch and Myrskylä (2009) observe no consistent relationship between income and support for the status quo of the public pension system. Some of these studies integrate only income or education and none of them consider financial literacy; considering all three factors together allows us to distinguish what exactly drives the result. Moreover, these studies draw on relatively old data and given that non-state pension pillars have expanded in recent decades, the alignment between income and education and the preferred pension mix may have changed, too.

Age also shapes material self-interest concerning pension preferences. Younger individuals are inclined toward non-state pensions (Boeri et al., 2001; Gelissen, 2001; van Groezen et al., 2009) for their flexibility and potentially higher returns. Occupational and private pensions are usually capital-funded, so contributions are invested in financial markets and future income depends on market developments. Individuals with a long-time horizon for such investments can thus obtain higher total returns than individuals with a brief time horizon for those investments as risks and losses can be offset (Browning, 1975). Furthermore, younger persons have higher risk tolerance, so they are more likely to prefer financial market investments (Brooks et al., 2018; Nicholson et al., 2005).

Older individuals, in contrast, tend to favour public pensions. This preference is driven by the desire for security and predictability in retirement income. As retirement approaches, the stability and reliability of public pensions, often managed by the government or social partners (unions and employer associations), become more attractive. Additionally, public pensions are less vulnerable to market fluctuations, which is a crucial consideration for those nearing retirement age. More generally, unlike in the case of occupational and individual private programmes, public pensions are usually financed through pay-as-you-go schemes and increases in public pension benefits are particularly advantageous for older generations (pensioners).

Gender differences also influence pension attitudes. Women are more likely to experience career interruptions and lower lifetime earnings, so they often favour public pensions (Ebbinghaus and Naumann, 2020; Frericks et al., 2009; Gelissen, 2001; Möhring, 2018) and are less supportive of private (Boeri et al., 2001; Jaime-Castillo, 2013) and occupational pensions (van Groezen et al., 2009). Public basic pension schemes, such as in the Netherlands and Denmark, account for such life course events that may have a negative impact on households’ financial situation by calculating pension benefits on years of residence in a country rather than employment careers (OECD, 2023). Men typically have higher incomes and continuous career trajectories compared to women, so they tend to prefer individual and occupational pensions. Moreover, women tend to live longer than men, increasing their need for a reliable income stream throughout retirement, which public pensions better ensure. Based on the previous discussion, we formulate the following hypotheses:

H1 High-income earners are associated with preferences for a larger pension income share of occupational and individual pension plans than middle- and low-income earners.

H2 Older persons have a stronger preference for large shares of public pension income than young persons.

H3 Women have a stronger preference for large shares of public pension income than men.

### Political ideology

Beyond socio-demographic factors, political ideology influences public pension preferences (Lynch and Myrskylä, 2009). Right-leaning individuals favour occupational and private pensions (Boeri et al., 2001; Gelissen, 2001; Jaime-Castillo, 2013), whilst their left-leaning counterparts support greater government involvement in the provision of retirement income. We assume that ideological predispositions develop before specific policy preferences, because political attitudes tend to form during adolescence and early adulthood (Jost et al., 2009), remaining stable afterwards (Stoker and Jennings, 2008).

The (socio-economic) left-right ideological spectrum should shape preferences regarding the pension income mix through beliefs about the role of government, market efficiency, and social welfare, similar to their effect on redistribution (Jæger, 2008). Individuals with left-leaning ideologies typically prefer public pensions, because they support a more substantial government role in providing social security and welfare services, including pensions. They give particular salience to redistribution, viewing public pensions as a means to ensure fair income distribution in retirement (Reeskens and van Oorschot, 2013). Furthermore, they are sceptical about the efficiency and fairness of market-based solutions, leading to a preference for public pensions over non-public pension income sources.

While occupational and individual pension schemes are often categorised as non-public pension pillars, they differ in important ways. Occupational pensions are often collectively organised by social partners, and, in some countries, they are quasi-mandatory elements of sectoral agreements. In contrast, individual pension plans are voluntary, market-based savings schemes where individuals bear investment risks. However, both occupational and private pensions rely on market mechanisms, at least to some extent, by investing contributions on financial markets. This is very different to the pay-as-you-go logic of public pension schemes (Anderson, 2025).

In contrast, individuals with right-leaning ideologies tend to favour occupational and individual pensions, because of the belief in the efficiency of market-based solutions compared to public ones. They prefer a lean welfare state and a limited government intervention in economic matters, so that lower (payroll) taxes leave more financial resources to individuals (Feldman and Zaller, 1992).

H4 Right-wing individuals have a higher preference for occupational and individual pension income shares than left-wing individuals.

### Feedback effects and institutions

A key finding of institutional theory is that social policies generate positive self-reinforcing feedback effects (Brooks and Manza, 2006; Busemeyer et al., 2021; Campbell, 2012; Pierson, 1993) among individuals whose main or second main source of income will be a private or occupational pension scheme showing preferences for these pension schemes (van Groezen et al., 2009). Social policies create enduring patterns of support based on resource effects related to material self-interest and they encourage the formation of support coalitions that promote the stability and/or expansion of the social policy in question.

Social policies that provide important benefits and require long periods of benefit accrual – such as all types of pension schemes – are most likely to generate positive feedback. Pension schemes typically require several years – public schemes up to 30 or 40 years of participation – in order to qualify for benefits or to receive adequate benefits. Moreover, the costs of shifting to a different pension mix or different pension products are high. Therefore, an individual’s reliance on a pension scheme increases its support.

H5 People with an occupational pension plan (individual pension plan) have a stronger preference for pension income from occupational (individual) pension plans than those without such a plan.

The broad coverage of pension systems generates strong political support for it because almost everyone has a vested interest in the system’s stability and longevity (Brooks and Manza, 2006; Campbell, 2012). Path dependence makes matured pension pillars particularly resistant to radical changes or reforms (Myles and Pierson, 2001) because people’s expectations and future plans are closely tied to the system’s continuation. In fact, the passage of any type of pension reform (expansionary or contracting) increases public salience (Fernández et al., 2023).

The structure of existing pension systems significantly influences individual (status quo) preferences (Boeri et al., 2001; Lynch and Myrskylä, 2009; van Groezen et al., 2009). Countries with well-established public pension systems (i.e., high replacement rates and low levels of private pension) tend to have populations more supportive of public solutions (Gelissen, 2001). In our cases, the design of the public-private pension mix might be decisive. The Netherlands, Denmark, and Ireland have flat-rate public pension schemes in the tradition of Beveridge that ensure a minimum income in old age (OECD, 2023). However, people need additional income from other pension pillars, especially occupational pension schemes, to ensure a decent living standard. In contrast, the public pension pillar in Austria, Germany, and Spain follows the Bismarckian social insurance path providing generous benefits solely based on the public pension pillar (although declining recently).

H6 People prefer a higher pension income share from occupational pensions in the Netherlands, Denmark, and Ireland, whilst people are in favour of high public pension benefits in Austria, Germany, and Spain.

## Data and methods

### Data

We test our hypotheses with the DEEPEN database, a novel cross-national online survey on individual experiences and attitudes towards occupational pension plans in six countries (Austria, Denmark, Germany, Ireland, the Netherlands and Spain) that also contains questions on pension systems in general, fielded by YouGov in April 2023. The samples were randomly selected from national web panels, with stratified sampling used to match the population proportions of age, education, gender, and geographical groups in each country and range from 1099 cases (Denmark) to 1484 (Spain). As often occurs with stratified samples, very small (1–2%) proportions of respondents corresponding to certain quotas do not complete the survey. To adjust for this, we apply weights in all descriptive and multivariate analyses. As a result, the samples are approximately representative of the population in the six countries. Since we are interested in individual preferences regarding the preferred future pension income mix our sample only consists of non-retired persons between 18 and 65 years.

The six countries represent different pension system configurations in affluent democracies. Earnings-related Bismarckian public pension schemes provide generous benefits in Austria, Germany and Spain. In contrast, universal flat-rate and Beveridge-type public pensions alone cannot provide status maintenance in retirement in Denmark, Ireland and the Netherlands so occupational pension schemes are very important (Immergut et al., 2007). Consequently, the six countries differ substantially in the relative size of private (occupational and individual) pension pillars. According to OECD (2023) data, occupational and individual pension plans represented 51.5%, 23.3% and 19.8% of old-age and survivor spending in the Netherlands, Ireland and Denmark, respectively. These proportions were only 6.3%, 5.1% and 3.4% in Germany, Austria and Spain, respectively.

### Variables

To operationalise preferences regarding the pension income mix we consider the three main sources of pension income: (i) public pension schemes; (ii) occupational pension plans organised via employers and financed with contributions of employers and/or employees; and (iii) voluntary individual pension plans. The questionnaire item asks “If you could choose your (future) mix of pension income, what would be your preference? Please, note that the sum of the three items must equal 100%.” Respondents then had three items with sliders (“public,” “occupational” and “private individual”), with each ranging from 0% to 100%. Hence, the three income sources had a zero-sum relationship.^1^ To facilitate the interpretation of the results, we analyse these three items as separate, continuous variables. In robustness checks, we replicate the main results with an alternative dependent variable (with four categories) that classifies respondents’ answers based on their preferred dominating pension pillar (whether they prefer a pension income mix with at least half of all income coming from (a) public, (b) occupational, (c) individual pension schemes, or (d) a mix of all pillars without a dominating income source).

We test our hypotheses for self-interest with respondents’ age (age groups: 18–24, 25–34, 35–44, 45–54 and 55 or more), gender (dichotomous variable for *female*), and equivalised household *income* weighted by the number of household members. Ideology is measured with respondents’ left-right ideology (10-point scale).^2^ The models also include *left-right ideology squared* to capture potential curvilinear effects, as both extreme-left and extreme-right individuals may favour a stronger role for public pensions than centrists-leaning individuals. While left-wing individuals support public pensions for redistribution and solidarity (Reeskens and van Oorschot, 2013), far-right voters often back generous social consumption welfare policies when targeted towards in-groups, such as native-born citizens, while opposing market-based solutions (Busemeyer et al., 2022; Enggist and Pinggera, 2022). Two dichotomous variables for having (a) an *occupational pension plan* and (b) an *individual pension plan* test the hypothesis about feedback effects.

To reduce the risk of biases due to unobserved heterogeneity, all models control for *education* (dichotomous variable distinguishing respondents with university education from the rest), *risk aversion* and current *trade union membership* (yes/no). The principles of inflation, compound interest and financial risks are central to individual finances in general and are a baseline for financial decision-making such as in the case of pension saving decisions. Respondents with higher financial literacy should, therefore, be better able to assess the personal consequences of different pension income mixes. Following this logic, all models include an index variable for *financial literacy* based on three questionnaire items designed based on respondents’ knowledge of those three concepts (Lusardi and Mitchell, 2008).^3^ The Appendix provides the questions and responses used to operationalise each variable verbatim and Table A1 reports descriptive statistics of all dependent and independent variables.

### Methods

We estimate OLS pooled models (with country fixed effects) as well as separate models for the six countries. The Netherlands is the reference country in the pooled models because it represents a paradigmatic case of a multi-pillar pension system with a strong quasi-mandatory occupational pension pillar. Pooled models have the advantage of larger sample sizes and are thus less sensitive to outliers. They also allow us to identify cross-national differences by controlling for compositional factors such as the percentage of university-educated respondents. OLS models for individual countries, by contrast, allow us to assess whether associations between variables are consistent or heterogenous across the six countries. We estimate the main models without multiple imputation. Yet, several independent variables have non-negligible numbers of missing value.^4^ As a robustness test, we therefore utilise a multiple imputation technique to obtain estimates from missing values in independent variables and replicate the main models with these imputed values.^5^ Furthermore, multinomial regression models test the robustness for the alternative categorical dependent variable.

## Results

### Pension income mix preferences

**Figure 1.**
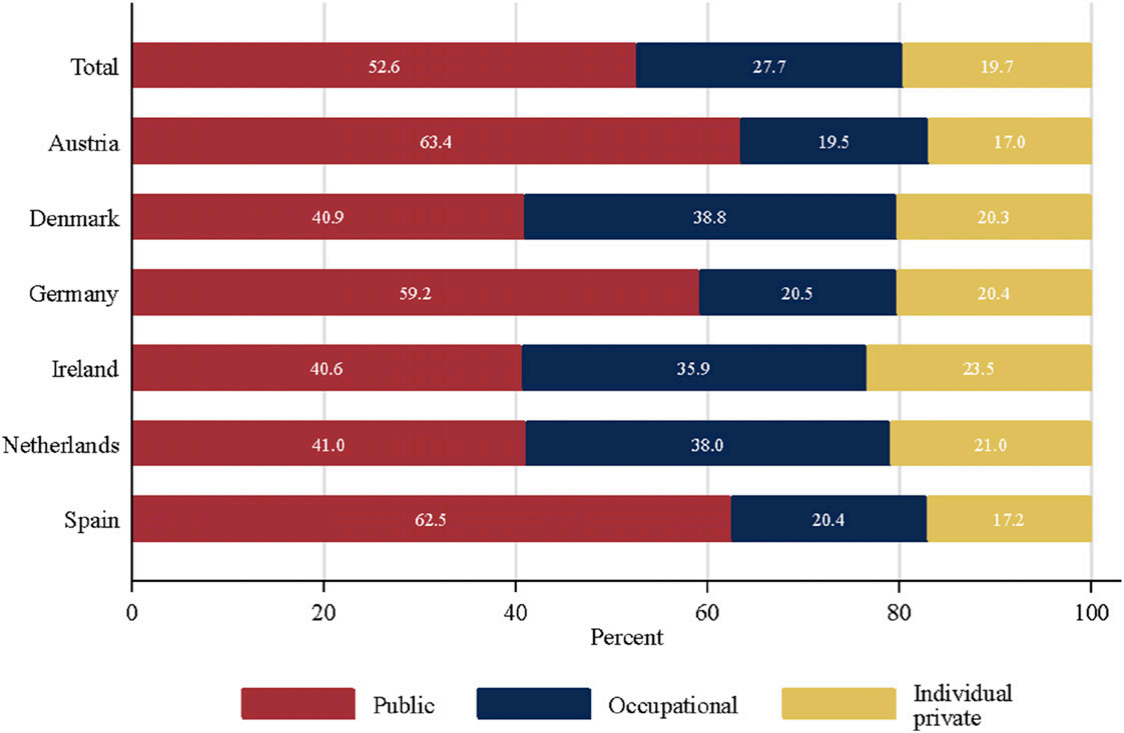
Average values in the preferred percentage of income from public, occupational and individual sources.

Figure 1 shows the average percentage of pension income respondents would like to have from (i) public, (ii) occupational, and (iii) individual pension schemes.^6^ The results clearly indicate that respondents prefer a combination of all three pension pillars rather than pension income from just one source. In the pooled sample, the preferred pension mix includes half of all income from public pensions (52.6%), around one-fourth from an occupational pension plan (27.7%) and one-fifth from an individual pension plan (19.7%).

The preferred income share from individual pension plans is similar across all countries (between 17.0% in Austria and 23.5% in Ireland), but we find substantial variation for the preferred income share from public and occupational pension schemes. The preferred pension mix shows a more homogeneous distribution in the three Beveridge countries – Denmark, Ireland and Netherlands – than in the three Bismarckian countries – Austria, Germany and Spain. In the latter three countries, respondents would like to have a pension mix with a dominant income share from public pension schemes (59–63%) and only one-fifth from an occupational pension plan. In contrast, people prefer a more equal share of pension income from public (41%) and occupational pension schemes (36–39%) in the three Beveridge countries.

However, public pension schemes should play a much more important role in retirement income for people in Bismarckian than in Beveridge countries. Furthermore, in all countries – except Germany – respondents prefer a higher income share from an occupational pension plan than an individual private one.

### Determinants of preferences

**Figure 2.**
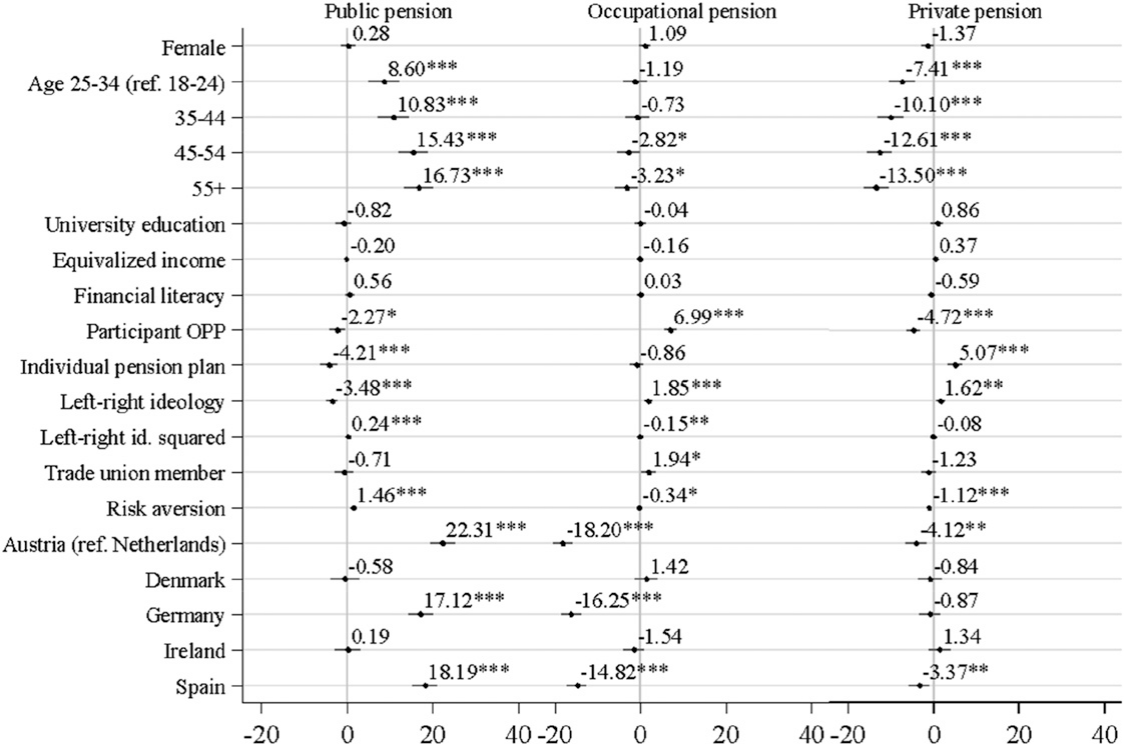
Linear models predicting the preferred proportion of public, occupational and private pension, pooled model. Note: +*p* < .1, *p < .05, **p < .01, ***p < .001.

Figure 2 (based on Table A2) presents the coefficients for three OLS models with individual determinants of the preferred percentage of each of the three pension income sources and five country fixed effects. Regarding self-interest, individuals with higher income show a higher preference for income from individual pension plans (significant only at the 0.1 level), but not from occupational pension plans. This partially supports H1 and is in line with similar findings by Gelissen (2001). The positive association of income with individual pensions likely reflects the need for additional savings due to public pension benefit caps. The lack of association with occupational pensions may be due to the inclusion of financial literacy and occupational and individual pension plan variables, which are more common among higher-income respondents.

**Figure 3.**
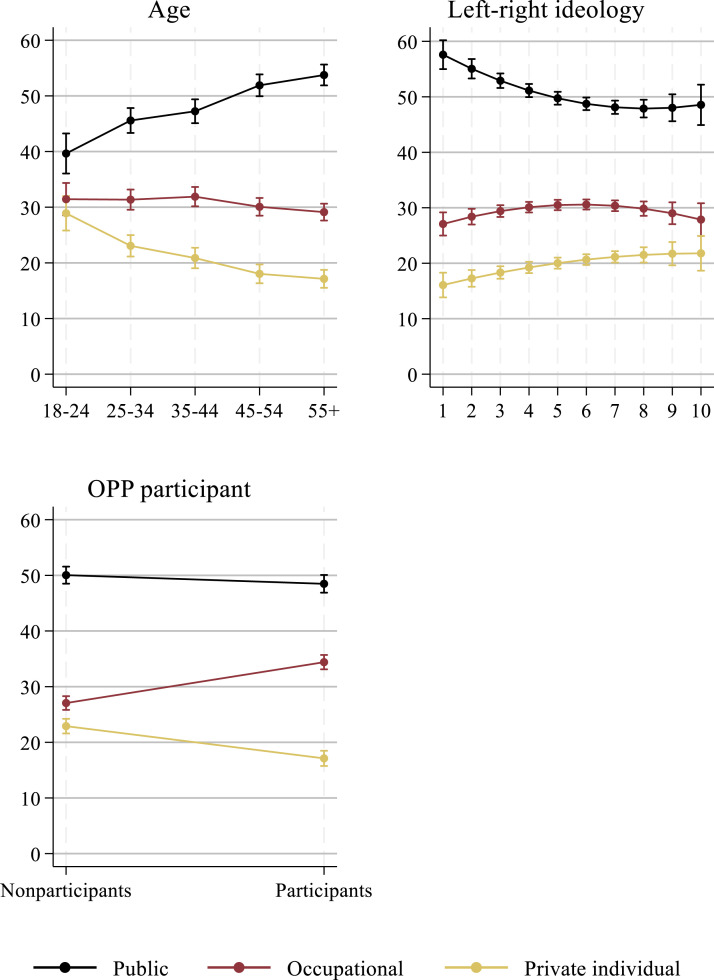
Predicted level in the three dependent variables at different levels in age, ideology and OPP participant (based on Table A2).

Age has a strong linear association with the three dependent variables. Consistent with H2, older respondents are significantly more likely to prefer a higher income share from public pension schemes and lower income share from occupational and private pension plans. The association is, moreover, especially significant in the case of public pensions. To assess the substantive effect of age, ideology and having an occupational plan, we estimate predicted values keeping all other variables at the average level and depict them in Figure 3. The preferred pension income mix of respondents 55-years-of-age or older has a 14.10 points higher proportion of income from public sources than the preferred pension income mix of respondents 18 to 24 years old. The gap is also substantial (although slightly smaller) regarding occupational and individual pension plans. The preferred proportion of income from individual pension plans of respondents 55 years old or older is 11.8 points lower than the proportion of respondents 18 to 24 years old (Figure 3). Age is thus a relevant determinant for pension income mix preferences. However, we acknowledge that we cannot identify a causal effect for age, because our cross-sectional analyses do not allow us to separate the effects of age, cohort, and period. The latter two might partially influence our age-related findings.^7^

In contrast to H2 we cannot confirm H3, because gender is not significantly related to any of the three outcomes, meaning women and men have similar pension income mix preferences, in line with van Groezen et al. (2009). Perhaps women have less information regarding these three types of schemes, so they do not recognise the advantages they would have from public pension schemes. Moreover, public debates about allegedly unsustainable public pension schemes might influence the preferences of men and women alike (Hagelund and Grødem, 2017; van den Heijkant et al., 2024). In sum, we find only limited support for self-interest as a predictor for pension income mix preferences.

In line with H4 and large parts of the literature (e.g. Gelissen, 2001; Jaime-Castillo, 2013; Lynch and Myrskylä, 2009), individuals’ political ideology is related to the preferred pension income mix. Respondents with a more right-leaning ideological outlook are significantly less likely to prefer a high proportion of public pension income and would rather have a significantly higher proportion of occupational and private pension income.

However, ideology displays a weak curvilinear association, because the squared term of the ideology variable is also significant, but with the opposite sign for public and occupational pension income share. Extremely politically left people show the highest preferred income share from public pension schemes (57.0%). This preference declines until a medium-high level of right political ideology (47.1%) and increases moderately again for extremely politically right people (48.6%) (Figure 3). A similar slight curvilinear pattern occurs for occupational pensions. The preference for a higher income share of occupational pension schemes increases when people move from the left to the right on the political ideology spectrum and peaks for people with a moderate right-wing political ideology. This suggests that strongly right-wing people have a higher preference for public pension income than people of the centre or modest right; and strongly right-wing persons have a lower preference for occupational pension income than people of the centre or modest right. These findings are in line with studies that suggest populist radical right parties and their voters are more sympathetic to social consumption policies such as (public) pensions than centrist parties and their voters (Busemeyer et al., 2022; Enggist and Pinggera, 2022).

The existing pension mix of a person is also a significant predictor for the preferred pension income mix, signalling feedback effects. Respondents with an occupational pension plan display a significantly higher average percentage of preferred occupational pension income and significantly lower average percentages of preferred public and private individual pension income. The association with occupational pension income is particularly substantial. Respondents with an occupational pension plan prefer a 7.4% points higher income share from occupational pension schemes than people without such a pension plan. Having an individual pension plan also proves relevant. Having such a plan is associated with an average 4.3% points higher preferred proportion of income from a private pension. These patterns support H5 and indicate that social policies create their own sources of support (Van Groezen et al., 2009).

Finally, we hypothesised that the country of residence shapes preferences concerning the pension income mix. Our results confirm previous findings (Gelissen, 2001; Lynch and Myrskylä, 2009; van Groezen et al., 2009) and show that, controlling for individual-level factors, people in Denmark, the Netherlands, and Ireland – Beveridge countries with public flat-rate pension systems – prefer a significantly higher pension income share from occupational pensions compared with Austria, Germany, and Spain. In contrast, the public pension pillar is expected to play a significantly larger role in Austria, Germany, and Spain – countries with a rather generous earnings-related Bismarckian public pension pillar – compared with the Netherlands, Denmark, and Ireland, supporting H6.^8^ Controlling for socio-demographic factors, respondents in Germany, for example, prefer a pension income mix characterised by 17.1% points higher income share from the public pension scheme and 16.3% points lower income share from occupational pension plans than respondents in the Netherlands.

Regarding the control variables, education and financial literacy prove unrelated to any of the three outcomes. Many studies investigating pension preferences include variables for either income, education, or financial literacy and therefore cannot distinguish what exactly drives the result. Considering all three factors together shows that other individual characteristics are better suited to predict pension income preferences.^9^ Moreover, our financial literacy measure captures general and not pension-specific financial knowledge, which may be more directly relevant for pension preferences. Similarly, controlling for individual ideology, trade union membership is unrelated to more public or lower individual pension income. Trade union members, however, prefer a slightly higher income share from occupational pensions, maybe because these rest on shared employer and employee contributions and joint employer/union administration in many countries. Risk aversion, in contrast, is consistently linked to the outcomes. Unsurprisingly, respondents who self-identify as risk-averse would like to receive pension income predominantly from a public pension scheme, and not from private – occupational or individual – plans.^10^

Pooled models may mask country differences. Therefore, we test the consistency of our five hypotheses (H1-5) for individual-level factors for each country separately.^11^ Figure A2 (and Tables A3–A8) shows that in all six countries – except Ireland and the Netherlands – age is strongly and positively related to the preference for public pension income. The fact that Ireland and the Netherlands have mature multi-pillar pension systems that provide substantial pension income from the three sources might explain this result. Age is negatively and significantly related to private individual pensions in all six countries. The lacking association for women and the preferred percentage of public pension income in the pooled model is less consistent across countries. Women prefer a higher (lower) income share from occupational pension plans in Austria and Denmark (Ireland) and a lower income share from individual pension plans in Austria and Germany. Income does not generally play a decisive role in pension income mix preferences except for the desire for a higher (lower) share of occupational pension income in Denmark (Spain) and higher (lower) public pension income in Spain (the Netherlands).

Right-wing political ideology is negatively related to the preference for public pension income for all six countries, but – possibly due to a small statistical power of the disaggregated tests – it is only significant for Germany, the Netherlands and Spain. Lastly, feedback effects for having an occupational pension plan found in the pooled model are confirmed in all countries except Austria and feedback effects for having an individual pension are confirmed in all countries except Germany and the Netherlands. This might be the result of very low rates of return in occupational pension plans in Austria over the last decade (Wiß, 2018) as well as for Riester pension plans in Germany (Nullmeier, 2021). Hence, once we consider the patterns for all six countries separately, some patterns are inconsistent which could be due to rather low sample sizes.

The results may be influenced by missing values in the independent variables and the operationalisation of the dependent variable. We thus test the robustness of our findings for the pooled model by (a) estimating the models with imputed missing values and (b) estimating a multinomial model for an alternative measure of the dependent variable. We replicate the main models in Figure 2 (and Table A2) with imputed values in independent variables (Table A9). The results are consistent with the findings from the main models reported above, with the only difference that *female* is now significant with opposite signs for the outcomes *occupational pensions* and *private individual pensions*. Women might value joint contributions by employers and employees and the joint administration of employers and trade unions in many countries for occupational pension plans.

Second, respondents may focus primarily on the income source they would like to dominate, rather than concrete percentages, when expressing their pension income preference. Therefore, in an additional analysis, we construct a categorical variable with four categories: preference for mostly public (50% or higher), mostly occupational (50% or higher), mostly private individual (50% or higher), and mixed pension income (<50% for each pension pillar). Table A10 shows the results of the multinomial regression models with “mostly public” as the reference category. On average, 57.2% of all respondents prefer a pension income mix in which the public pension scheme provides at least half of the pension income, 18.9% prefer a mostly occupational, 12.0% a mostly individual pension income mix, and 11.9% have no preference for a dominating pension pillar (mixed category).

The regression results confirm the main findings for the self-interest hypotheses: older respondents would like to have a smaller income share from individual private pensions, and gender is not a significant predictor. Politically right-leaning persons are more likely to prefer a pension income mix with a dominant role for individual pension plans, not occupational pension plans, and the associations are not curvilinear. The feedback effect for individual pension plans, however, disappears. Therefore, the relationship we find for individual pensions in the main models does not mean that they should dominate the pension income mix. Using this multinomial outcome, the country differences (H6) largely reflect the results of the main models with people in the Netherlands, Denmark and Ireland rather than in Austria and Germany being more likely to prefer a dominating role for occupational pension income. In sum, although this dependent variable measures something slightly different (the preferred dominating pension income source rather than the preferred income share of each pension pillar) the results are rather robust.

## Conclusion

The analysis presented here advances our understanding of public preferences regarding the income mix of public, occupational, and individual pensions in six European countries with distinct pension systems. Considering the preferred retirement income share from the three pension system pillars does justice to actual pension systems and individual income mixes of current and future pensioners in most European countries that consist of several pension income sources. Moreover, measuring preferences under current pension legislation is a much-needed update of studies that investigated similar questions with data for the 1990s and 2000s, before major reforms had been initiated and implemented. Our original online survey in six countries with different public-private mixes in their pension systems reveals that on average people value public pensions rather highly. The public prefers a pension income mix with slightly more than half of all income coming from public pensions schemes. Yet respondents in the six countries would also like to have a substantial share of the retirement income from occupational and individual pensions. In general terms, the preference is for an unbalanced pension mix with a predominant public pillar supplemented by income from occupational and individual pensions.

Our findings demonstrate that public preferences for the pension income mix are shaped by self-interest, ideology, and institutional context. These insights are particularly relevant for policymakers, as pension reforms typically require public support for successful implementation. The findings highlight the role of self-interest in shaping pension preferences. Older individuals tend to favour public pensions, likely due to the security and predictability they offer as retirement approaches. In contrast, younger people, who have a longer time horizon for investments and a higher tolerance for risk, show a preference for occupational and individual pensions. Interestingly, while income weakly influences preferences for individual pension plans, its impact on preferences for occupational pensions is less pronounced. This may suggest that the role of income is nuanced, potentially mediated by other factors such as financial literacy and existing pension plan participation.

Political ideology also emerges as a significant determinant of pension preferences, with right-leaning individuals generally favouring occupational and individual pensions, while left-leaning individuals prefer a stronger role for public pensions. However, the mild curvilinear relationship observed in some cases suggests that extreme political positions on both ends of the spectrum may have more in common than previously thought, particularly concerning support for public pensions. This finding contributes to a growing body of literature on the complex ways in which ideology shapes social policy preferences.

This study provides strong evidence of feedback effects, where individuals’ participation in specific pension schemes reinforces their preferences for income from those schemes. This finding aligns with institutional theories, which posit that social policies and organisational structures create their own constituencies. We can very likely exclude reverse causality – people who prefer larger income shares from occupational pension plans chose to have these plans –, because even in cases where occupational pension schemes are mandatory, as in the Netherlands and Denmark, the associations between having an occupational pension plan and a preferred larger income share from these schemes are significant. However, we recognize that some degree of self-selection might exist in pension schemes, particularly for individual pensions, where people who prefer private retirement savings may proactively choose to enrol in such plans. Moreover, preferences may reflect perceived accessibility, as those without occupational pensions may not oppose them but see them as unavailable. Additionally, feedback effects may stem from the cognitive complexity of pension systems, where individuals rely on familiar schemes rather than assessing alternatives they may not fully understand. Future research should disentangle these effects from informational constraints by distinguishing between preferred preferences and perceived access. The policy implication is clear: expanding access to occupational and individual pension plans could gradually shift public preferences toward a more mixed pension income model. However, policymakers must consider the potential for resistance from those who are already heavily reliant on public pensions and may view such reforms as threatening their financial security.

Finally, we identify a very strong influence of the existing pension system on public preferences. In countries with a strong tradition of Bismarckian public pension systems (Austria, Germany, and Spain), there is a marked preference for a higher share of public pension income. Conversely, in countries with Beveridge-type flat-rate pension systems (Denmark, Ireland, and the Netherlands), the public shows a stronger inclination towards a balanced mix, with occupational pensions playing a more significant role. This underscores the importance of the institutional context in shaping public attitudes towards pension systems and suggests that pension reforms must be carefully tailored to specific national institutional and cultural contexts to be effective and widely accepted. Overall, we show that institutional arguments have the strongest explanatory power for pension income preferences, followed by self-interest (individuals’ age).

Our survey covers only a limited number of countries and future research could extend the analysis to further countries. Moreover, exploring how these preferences evolve over time, particularly in response to changes in the economic environment and pension policies is a promising future research endeavour. In conclusion, this study offers valuable insights for policymakers seeking to reform pension systems in Europe. Understanding public preferences is crucial for designing reforms that are both effective and politically feasible.

## Supplemental Material


Supplemental Material - Attitudes towards the public-private mix for retirement income in Europe
Supplemental Material for Attitudes towards the public-private mix for retirement income in Europe by Tobias Wiß, Juan J Fernández and Karen M Anderson in Journal of European Social Policy.

## Data Availability

Replication materials for the paper can be found in: https://osf.io/z4xes/?view_only=9bab7dbb61504932a4413b919734bd72.

## Notes

1. A drawback for many studies analysing preferences is that respondents may consider the actual availability of (pension) options rather than purely hypothetical preferences, though we ask them in a hypothetical way. This ‘realistic conditioning’ may vary across groups in our case: while it does not affect mandatory public, mandatory occupational, or voluntary individual pensions, those without access to occupational pensions may be less likely to choose them, not due to preference but perceived inaccessibility.

2. While the left-right scale is widely used in attitudinal research, it may not fully capture political complexity with regard to the socio-cultural dimension. Despite this, it remains the most common and meaningful measure across different institutional contexts (Jæger, 2008). The economic left-right dimension is particularly relevant for pensions and social policy as it reflects fundamental differences in support for state versus market-based welfare provision. Moreover, it has proven useful albeit not being perfect in predicting welfare and pension attitudes (Reeskens and van Oorschot, 2013).

3. Only 39% of all respondents answer all three items correctly.

4. Table A11 includes the percent missing in all independent variables.

5. We specifically estimate 50 imputed values for each independent variable using chained equations and models in Stata and including the ‘force’ option. Missing values in the dependent variables were not imputed.

6. Figure A1 in the Appendix provides the same percentages with confidence intervals.

7. Conceptualising age from a cohort, rather than biographical, perspective could point to a somewhat different interpretation. Many younger cohorts have experienced public pension retrenchment, as well as expansion of occupational and private pensions. Younger cohorts may, for instance, be more tolerant of financial risk due to the institutional and policy landscape of their formative years. Our data is cross-sectional, so we cannot distinguish between age and cohort effects. Future research using longitudinal data could track how individual pension preferences evolve with age.

8. Predicted values for each of the six countries in each of the three outcomes are available upon request.

9. The correlations between these three factors range from 0.145 to 0.249.

10. Additional analyses (available upon request) indicate that respondents’ marital and employment statuses are unrelated to preferences for public and individual pensions, whilst all other variables maintain the same associations. Respondents in employment (vs not employed) are significantly less and married/with partner (vs single) are more likely to prefer a higher proportion of occupational pension income. However, these models do not include Spain and therefore decrease sample sizes, as there is no data on marital status for this country.

11. We estimate 18 regression models: one for each of the three pension pillars for each of the six countries (Tables A3–A8).
