# Supplementary material for: Attitudes towards the public-private mix for retirement income in Europe
Source: J Eur Soc Policy. 2025 Jun 3;36(1):51–66. doi: 10.1177/09589287251345904 (PMC12822915; doi:10.1177/09589287251345904)
Supplement: Supplemental Material - Attitudes towards the public-private mix for retirement income in Europe [file sj-pdf-1-esp-10.1177_09589287251345904.pdf]

Appendix

Wiß, Tobias, Fernández, Juan J and Anderson, Karen M (2025): Attitudes towards the public-private mix for retirement income in Europe. Journal of European Social Policy.

Definition and codification of all variables

Preferences for the pension mix: "If you could choose your (future) mix of pension income, what would be your preference? Please, note that the sum of the three items must sum 100%." Items: "Public pension", "Occupational pension" and "Private individual pension".

Female: We utilize a question already included in the YouGov national panels. It distinguishes respondents who are female (1) from those who are male (0).

Age: We utilize a question regarding respondent's age already included in the YouGov national panels. For the analysis we distinguish five age groups: respondents aged 18-24 (1), 25-34 (2), 35-44 (3), 45-54 (4) and 55 or more (5).

University education: This variable distinguishes respondents who completed a university degree (B.A., M.A, PhD) (1) from other respondents (0).

Financial literacy: Following Lusardi and Mitchel (2008), we utilize three knowledge questions. The first one asks "Suppose you had 100€ in a savings account and the interest rate was 2% per year. After 5 years, how much do you think you would have in the account if you left the money to grow?" with response options "More than 102€" (1), "Exactly 102€" (2), "Less than 102€" (3). The second one asks "Imagine that the interest rate on your savings account was 1% per year and inflation was 2% per year. After 1 year, how much would you be able to buy with the money in this account?" with response options "More than today" (1), "Exactly the same" (2) and "Less than today" (3). The third one asks "Please indicate whether this statement is true or false. Buying a single company's stock usually provides a safer return than a stock mutual fund." with response options "True" (1) and "False" (2). Correct answers are (1), (3) and (2). We assign a value of 1 to all correct answers. "Don't know" responses are coded as (0).

Equivalized income: Respondents are asked "Using the following brackets, please indicate which letter describes your household's total weekly income, after tax and compulsory deductions, from all sources? If you don't know the exact figure, please give an estimate." They are then provided 10 brackets corresponding to approximately 10 income deciles in the country. The values were obtained from round 9 of European Social Survey. The values were then updated with the average CPI value (Eurostat 2023).

Trade union member: The question reads "Are you or have you ever been a member of a trade union or a professional association?" with response options "Yes, currently", "Yes, previously" and "No". The variable distinguishes current members (1) from the rest (0).

Left-right ideology: The question reads "In politics people sometimes talk about "left" and "right". Where would you place yourself on this scale, where 0 means the left and 10 means the right?" with response options ranging from 0-10.

OPP participant: We identify respondents who are participants in OPP through their responses to four questions. The first question asks to the person if they are retired or not. "Are you retired?" Response options are "Yes" and "No". The second question asks respondents "Which of these descriptions applies to what you have been doing for the last 7 days?" The key response option is "in paid work (or away temporarily) (employee, self-employed, working for a family business)". The third question asks respondents "Does your current employment offer an occupational pension plan? By occupational pension plan we mean pensions and other monetary transfers for old-age and disability linked to

your employment and aimed at supplementing state-based pensions. (We do not refer to individual pension plans you may have.)". Response options: "Yes", "No". The fourth question asks "Are you a member of this occupational pension plan?". Response options: "Yes", "No". We identify as participants (1) all respondents who are not retired, are employed, their employment offer an occupational pension plan and they are member of it.

Individual private pension: Dichotomous variable indicating whether the respondent has an individual pension plan. The original questionnaire item reads "Which of these financial products, if any, do you currently hold money or invest in (alongside your occupational pension plan)?". This financial asset is "An individual pension plan".

Risk aversion: Level of risk aversion of respondents. The original questionnaire item reads "Are you generally a person who is willing to take risk or do you try to avoid taking risks? Please tick a box on the scale, where the value 0 means not at all willing to take risks and the value 10 means very willing to take risks." Response options range from "Very risk averse" (0) and "Very willing to take risks" (10). We reverse coded the variable to "Very willing to take risks" (0) and "Very risk averse" (10).

Tables and figures

Table A1. Descriptive statistics of all variables (weighted)

Variable	Obs	Mean	Std. dev.	Min	Max
Public	4,286	52.601	27.271	0.000	100.000
Occupational	4,286	27.690	21.247	0.000	100.000
Private individual	4,286	19.710	21.437	0.000	100.000
Female	7,563	0.497	0.500	0.000	1.000
Age 18-24	7,563	0.127	0.333	0.000	1.000
Age 25-34	7,563	0.203	0.402	0.000	1.000
Age 35-44	7,563	0.210	0.408	0.000	1.000
Age 45-54	7,563	0.225	0.418	0.000	1.000
Age 55+	7,563	0.234	0.424	0.000	1.000
University education	7,322	0.407	0.491	0.000	1.000
Equivalized income	6,058	3.546	1.871	0.354	10.000
Financial literacy	7,229	2.013	0.972	0.000	3.000
Participant in OPP	6,839	0.339	0.473	0.000	1.000
Individual pension plan	7,563	0.162	0.368	0.000	1.000
Left-right ideology	6,835	5.135	2.260	0.000	10.000
Left-right ideology squared	6,835	31.480	24.077	0.000	100.000
Trade union membership	7,150	0.236	0.425	0.000	1.000
Risk aversion	7,162	5.049	2.630	0.000	10.000

Figure A1. Average values in the preferred percentage of income from public, occupational and individual sources

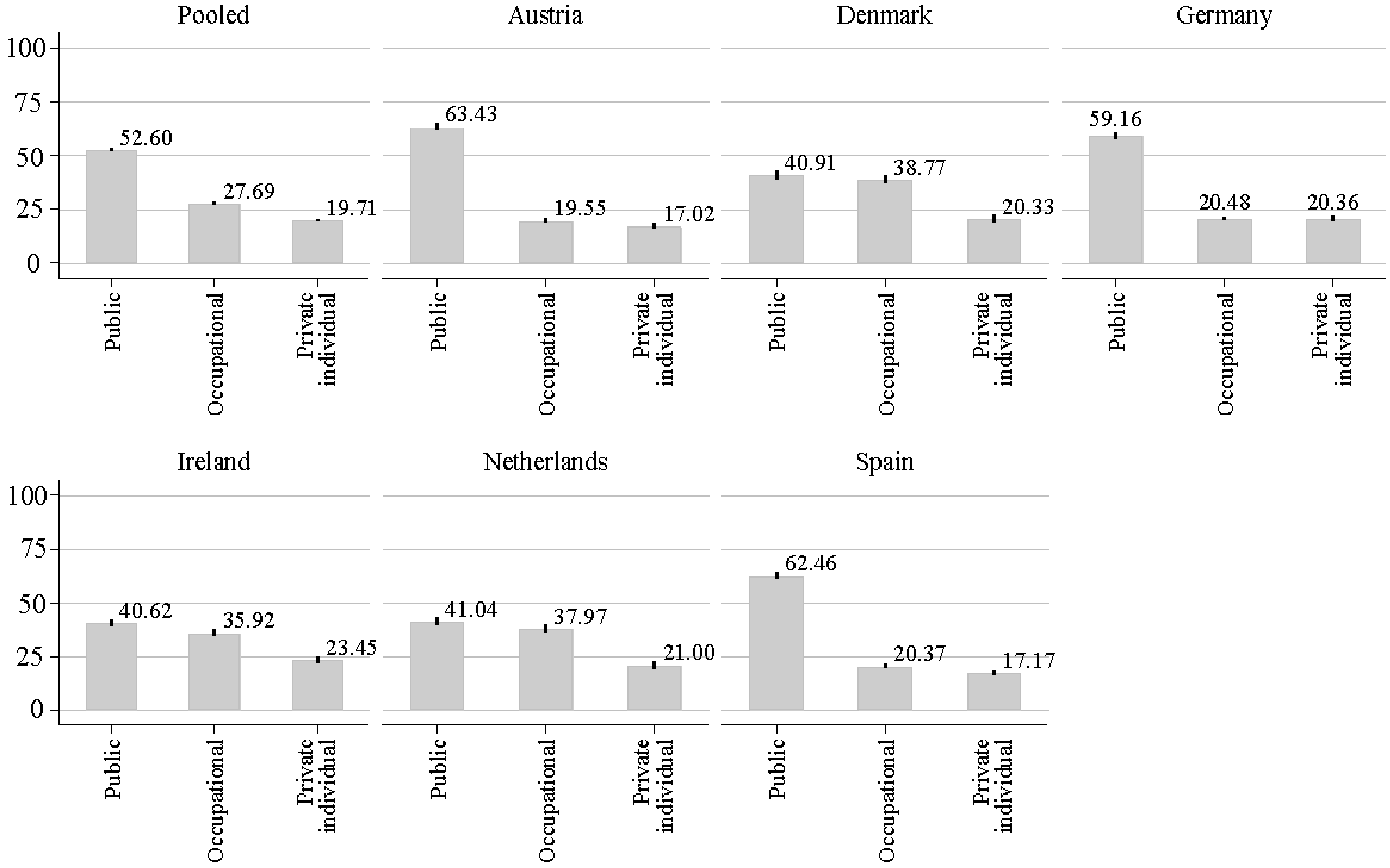

Note: Vertical bars represent 95% confidence intervals

Table A2. OLS models predicting the preferred percentage of income from the three sources, Pooled model

	Model 1	Model 2	Model 3
	Public	Occupational	Private individual
Female	0.279	1.088	-1.367+
	(0.859)	(0.672)	(0.718)
Age 25-34 (ref. 18-24)	8.605***	-1.191	-7.414***
	(1.850)	(1.448)	(1.546)
Age 35-44	10.825***	-0.727	-10.099***
	(1.834)	(1.436)	(1.533)
Age 45-54	15.433***	-2.821*	-12.612***
	(1.784)	(1.396)	(1.491)
Age 55+	16.732***	-3.228*	-13.503***
	(1.787)	(1.398)	(1.493)
University education	-0.825	-0.039	0.864
	(0.893)	(0.699)	(0.746)
Equivalized income	-0.201	-0.164	0.365+
	(0.254)	(0.199)	(0.213)
Financial literacy	0.560	0.029	-0.589
	(0.546)	(0.427)	(0.456)
Participant OPP	-2.267*	6.990***	-4.723***
	(0.947)	(0.741)	(0.792)
Individual pension plan	-4.208***	-0.858	5.066***
	(1.042)	(0.816)	(0.871)
Left-right ideology	-3.479***	1.855***	1.624**
	(0.693)	(0.542)	(0.579)
Left-right ideology squared	0.235***	-0.154**	-0.082
	(0.065)	(0.051)	(0.055)
Trade union member	-0.707	1.940*	-1.233
	(1.085)	(0.850)	(0.907)
Risk aversion	1.463***	-0.342*	-1.121***
	(0.170)	(0.133)	(0.142)
Austria (ref. Netherlands)	22.312***	-18.196***	-4.116**
	(1.526)	(1.195)	(1.276)
Denmark	-0.575	1.419	-0.843
	(1.762)	(1.379)	(1.473)
Germany	17.120***	-16.247***	-0.873
	(1.512)	(1.184)	(1.264)
Ireland	0.193	-1.537	1.344
	(1.545)	(1.209)	(1.291)
Spain	18.188***	-14.818***	-3.370**
	(1.518)	(1.188)	(1.268)
Constant	34.656***	32.993***	32.351***
	(2.963)	(2.320)	(2.476)
Observations	3,325	3,325	3,325
R-squared	0.223	0.222	0.098

Note: Standard errors in parentheses; *** p<0.001, ** p<0.01, * p<0.05, + p<0.1

Table A3. OLS models predicting the preferred percentage of income from the three sources, Austria			
	Model 1	Model 2	Model 3
	Public	Occupational	Private individual
Female	0.577	2.510*	-3.087*
	(2.014)	(1.274)	(1.520)
Age 25-34 (ref. 18-24)	8.887*	-0.107	-8.780**
	(3.835)	(2.427)	(2.896)
Age 35-44	12.724**	-1.565	-11.159***
	(3.952)	(2.502)	(2.984)
Age 45-54	18.473***	-5.054*	-13.419***
	(3.768)	(2.385)	(2.845)
Age 55+	21.882***	-4.828*	-17.054***
	(3.768)	(2.385)	(2.845)
University education	-0.695	0.642	0.053
	(2.059)	(1.303)	(1.555)
Equivalized income	0.529	-0.545	0.017
	(0.546)	(0.345)	(0.412)
Financial literacy	-0.167	-0.054	0.221
	(1.237)	(0.783)	(0.934)
Participant OPP	4.755*	0.319	-5.074***
	(1.948)	(1.233)	(1.471)
Individual pension plan	-7.582***	2.779+	4.803**
	(2.265)	(1.433)	(1.710)
Left-right ideology	-2.301	1.116	1.186
	(1.576)	(0.998)	(1.190)
Left-right ideology squared	0.078	-0.072	-0.007
	(0.147)	(0.093)	(0.111)
Trade union member	-3.286	1.451	1.835
	(2.089)	(1.322)	(1.577)
Risk aversion	1.017**	-0.356	-0.661*
	(0.388)	(0.245)	(0.293)
Constant	52.755***	19.932***	27.313***
	(6.294)	(3.983)	(4.752)
Observations	589	589	589
R-squared	0.145	0.053	0.145

Note: Standard errors in parentheses; *** p<0.001, ** p<0.01, * p<0.05, + p<0.1

Table A4. OLS models predicting the preferred percentage of income from the three sources, Denmark

	Model 1	Model 2	Model 3
	Public	Occupational	Private individual
Female	-5.235+	6.347*	-1.112
	(2.736)	(2.520)	(2.295)
Age 25-34 (ref. 18-24)	3.967	-1.556	-2.411
	(5.341)	(4.919)	(4.480)
Age 35-44	10.092+	-1.613	-8.479+
	(5.300)	(4.881)	(4.445)
Age 45-54	15.212**	-6.711	-8.501+
	(5.179)	(4.770)	(4.344)
Age 55+	16.755**	-10.168*	-6.587
	(5.247)	(4.832)	(4.401)
University education	-6.291*	3.773	2.518
	(2.755)	(2.537)	(2.310)
Equivalized income	-1.138	1.484*	-0.346
	(0.734)	(0.676)	(0.616)
Financial literacy	-0.621	0.503	0.117
	(1.848)	(1.702)	(1.550)
Participant OPP	-5.242+	7.096*	-1.854
	(2.994)	(2.758)	(2.512)
Individual pension plan	-0.842	-3.735	4.577*
	(2.699)	(2.486)	(2.264)
Left-right ideology	-3.268+	1.141	2.127
	(1.942)	(1.789)	(1.629)
Left-right ideology squared	0.253	-0.083	-0.170
	(0.182)	(0.167)	(0.152)
Trade union member	-3.145	5.000*	-1.855
	(2.742)	(2.525)	(2.300)
Risk aversion	1.912***	-0.453	-1.459**
	(0.560)	(0.516)	(0.470)
Constant	47.076***	24.785***	28.139***
	(7.855)	(7.235)	(6.589)
Observations	386	386	386
R-squared	0.164	0.130	0.088

Note: Standard errors in parentheses; *** p<0.001, ** p<0.01, * p<0.05, + p<0.1

Table A5. OLS models predicting the preferred percentage of income from the three sources, Germany

	Model 1	Model 2	Model 3
	Public	Occupational	Private individual
Female	2.932	1.271	-4.203*
	(2.026)	(1.252)	(1.748)
Age 25-34 (ref. 18-24)	13.561***	-3.359	-10.202**
	(4.047)	(2.500)	(3.491)
Age 35-44	16.010***	-5.312*	-10.698**
	(4.058)	(2.507)	(3.501)
Age 45-54	23.623***	-6.058*	-17.565***
	(3.975)	(2.456)	(3.429)
Age 55+	25.166***	-6.408**	-18.758***
	(3.806)	(2.351)	(3.283)
University education	-0.599	0.567	0.032
	(2.020)	(1.248)	(1.742)
Equivalized income	-0.403	-0.469	0.872+
	(0.532)	(0.329)	(0.459)
Financial literacy	0.500	0.848	-1.348
	(1.309)	(0.808)	(1.129)
Participant OPP	-1.826	7.137***	-5.311**
	(2.082)	(1.286)	(1.796)
Individual pension plan	-1.346	-1.239	2.585
	(2.209)	(1.365)	(1.906)
Left-right ideology	-5.459*	0.423	5.036*
	(2.372)	(1.465)	(2.046)
Left-right ideology squared	0.422+	-0.015	-0.408*
	(0.219)	(0.135)	(0.189)
Trade union member	2.891	1.376	-4.267+
	(2.975)	(1.838)	(2.566)
Risk aversion	1.958***	-0.318	-1.640***
	(0.387)	(0.239)	(0.334)
Constant	45.828***	21.950***	32.222***
	(7.960)	(4.918)	(6.867)
Observations	586	586	586
R-squared	0.168	0.076	0.173

Note: Standard errors in parentheses; *** p<0.001, ** p<0.01, * p<0.05, + p<0.1

Table A6. OLS models predicting the preferred percentage of income from the three sources, Ireland			
	Model 1	Model 2	Model 3
	Public	Occupational	Private individual
Female	1.354	-3.781*	2.427
	(2.014)	(1.821)	(1.870)
Age 25-34 (ref. 18-24)	-1.441	7.338+	-5.897
	(4.420)	(3.998)	(4.105)
Age 35-44	-4.368	10.363**	-5.995
	(4.287)	(3.877)	(3.981)
Age 45-54	1.230	7.211+	-8.440*
	(4.317)	(3.904)	(4.009)
Age 55+	5.689	2.230	-7.919+
	(4.476)	(4.048)	(4.156)
University education	1.582	-0.050	-1.532
	(2.197)	(1.987)	(2.040)
Equivalized income	-0.494	-0.344	0.837
	(0.666)	(0.603)	(0.619)
Financial literacy	-1.875	1.930+	-0.055
	(1.234)	(1.116)	(1.146)
Participant OPP	-2.087	11.113***	-9.026***
	(2.294)	(2.075)	(2.131)
Individual pension plan	-1.540	-4.280+	5.820*
	(2.624)	(2.373)	(2.437)
Left-right ideology	-1.298	1.049	0.249
	(1.361)	(1.231)	(1.264)
Left-right ideology squared	0.048	-0.113	0.065
	(0.140)	(0.127)	(0.130)
Trade union member	-0.018	1.537	-1.518
	(2.539)	(2.296)	(2.358)
Risk aversion	0.619	0.419	-1.038*
	(0.433)	(0.391)	(0.402)
Constant	49.064***	19.827***	31.109***
	(5.851)	(5.291)	(5.433)
Observations	593	593	593
R-squared	0.045	0.120	0.094

Note: Standard errors in parentheses; *** p<0.001, ** p<0.01, * p<0.05, + p<0.1

Table A7. OLS models predicting the preferred percentage of income from the three sources, Netherlands			
	Model 1	Model 2	Model 3
	Public	Occupational	Private individual
Female	2.759	-1.301	-1.458
	(2.532)	(2.337)	(2.398)
Age 25-34 (ref. 18-24)	7.331	-2.556	-4.775
	(5.674)	(5.236)	(5.374)
Age 35-44	10.226+	-0.855	-9.371+
	(5.542)	(5.114)	(5.249)
Age 45-54	7.472	1.869	-9.341+
	(5.421)	(5.003)	(5.134)
Age 55+	6.253	4.675	-10.928*
	(5.406)	(4.989)	(5.120)
University education	1.837	-3.791+	1.954
	(2.489)	(2.296)	(2.357)
Equivalized income	-1.396*	0.733	0.663
	(0.681)	(0.628)	(0.645)
Financial literacy	0.426	-1.098	0.673
	(1.662)	(1.534)	(1.575)
Participant OPP	0.177	6.646**	-6.823**
	(2.443)	(2.255)	(2.314)
Individual pension plan	-0.902	1.027	-0.125
	(4.031)	(3.720)	(3.818)
Left-right ideology	-3.790*	3.207+	0.583
	(1.895)	(1.749)	(1.795)
Left-right ideology squared	0.304+	-0.282+	-0.021
	(0.178)	(0.164)	(0.169)
Trade union member	-0.927	3.337	-2.410
	(3.393)	(3.131)	(3.213)
Risk aversion	1.203*	-0.162	-1.041*
	(0.538)	(0.497)	(0.510)
Constant	42.549***	27.272***	30.179***
	(8.220)	(7.586)	(7.786)
Observations	444	444	444
R-squared	0.053	0.066	0.065

Note: Standard errors in parentheses; *** p<0.001, ** p<0.01, * p<0.05, + p<0.1

Table A8. OLS models predicting the preferred percentage of income from the three sources, Spain			
	Model 1	Model 2	Model 3
	Public	Occupational	Private individual
Female	-1.048	1.946	-0.898
	(1.922)	(1.317)	(1.463)
Age 25-34 (ref. 18-24)	17.346***	-6.105*	-11.241***
	(4.433)	(3.037)	(3.373)
Age 35-44	19.757***	-5.157+	-14.601***
	(4.336)	(2.971)	(3.299)
Age 45-54	24.418***	-8.571**	-15.847***
	(4.116)	(2.820)	(3.131)
Age 55+	25.125***	-8.020**	-17.105***
	(4.217)	(2.889)	(3.208)
University education	-2.759	0.852	1.906
	(1.943)	(1.331)	(1.478)
Equivalized income	1.669*	-1.759***	0.090
	(0.671)	(0.459)	(0.510)
Financial literacy	4.449***	-2.514**	-1.935*
	(1.147)	(0.786)	(0.873)
Participant OPP	-7.115**	6.137**	0.978
	(2.731)	(1.871)	(2.078)
Individual pension plan	-8.961***	1.302	7.658***
	(2.323)	(1.592)	(1.767)
Left-right ideology	-5.319***	2.360*	2.958*
	(1.593)	(1.091)	(1.212)
Left-right ideology squared	0.351*	-0.176+	-0.175
	(0.144)	(0.099)	(0.109)
Trade union member	3.788	-2.303	-1.485
	(2.687)	(1.841)	(2.044)
Risk aversion	1.356***	-0.597*	-0.758**
	(0.346)	(0.237)	(0.263)
Constant	38.799***	32.621***	28.580***
	(6.020)	(4.125)	(4.580)
Observations	727	727	727
R-squared	0.182	0.108	0.118

Note: Standard errors in parentheses; *** p<0.001, ** p<0.01, * p<0.05, + p<0.1

Table A9. OLS models predicting the preferred percentage of income from the three sources using multiple imputation, Pooled model

	Model 1	Model 2	Model 3
	Public	Occupational	Private individual
Female	-0.091	1.377*	-1.287*
	(0.761)	(0.604)	(0.643)
Age 25-34 (ref. 18-24)	7.545***	-1.150	-6.395***
	(1.580)	(1.253)	(1.333)
Age 35-44	10.931***	-1.574	-9.357***
	(1.558)	(1.235)	(1.314)
Age 45-54	14.443***	-2.707*	-11.735***
	(1.515)	(1.202)	(1.278)
Age 55+	16.672***	-3.831**	-12.842***
	(1.532)	(1.215)	(1.293)
University education	-1.089	0.685	0.404
	(0.813)	(0.642)	(0.679)
Equivalized income	-0.277	-0.187	0.464*
	(0.247)	(0.197)	(0.207)
Financial literacy	1.046*	-0.254	-0.793+
	(0.483)	(0.383)	(0.412)
Participant OPP	-1.515+	6.459***	-4.944***
	(0.853)	(0.675)	(0.724)
Individual pension plan	-4.696***	-0.316	5.011***
	(0.939)	(0.745)	(0.793)
Left-right ideology	-3.263***	1.493**	1.770***
	(0.624)	(0.494)	(0.529)
Left-right ideology squared	0.219***	-0.129**	-0.090+
	(0.059)	(0.047)	(0.050)
Trade union member	0.154	1.562*	-1.717*
	(0.978)	(0.780)	(0.828)
Risk aversion	1.586***	-0.394**	-1.191***
	(0.153)	(0.121)	(0.129)
Austria (ref. Netherlands)	22.047***	-17.938***	-4.109***
	(1.312)	(1.041)	(1.108)
Denmark	0.639	-0.817	0.177
	(1.538)	(1.222)	(1.299)
Germany	18.008***	-16.689***	-1.319
	(1.314)	(1.043)	(1.109)
Ireland	0.522	-2.657*	2.135+
	(1.352)	(1.073)	(1.142)
Spain	19.868***	-15.234***	-4.634***
	(1.314)	(1.043)	(1.110)
Constant	32.068***	35.789***	32.143***
	(2.559)	(2.035)	(2.176)
Observations	4,286	4,286	4,286

Note: Standard errors in parentheses; *** p<0.001, ** p<0.01, * p<0.05, + p<0.1

Table A10. Multinomial model predicting the pension mix (ref. mostly public), Pooled model

	Mostly public (average: 57.2%)	Occupational (average: 18.9%)	Individual private (average: 12.0%)	Mixed (average: 11.9%)
Female		0.090	-0.165	-0.187
		(0.105)	(0.124)	(0.120)
Age 25-34 (ref. 18-24)		-0.253	-0.728**	-1.029***
		(0.252)	(0.224)	(0.222)
Age 35-44		-0.039	-0.863***	-1.085***
		(0.247)	(0.226)	(0.222)
Age 45-54		-0.440+	-1.220***	-1.392***
		(0.244)	(0.223)	(0.217)
Age 55+		-0.618*	-1.502***	-1.739***
		(0.247)	(0.228)	(0.226)
University education		0.100	0.048	0.049
		(0.111)	(0.127)	(0.124)
Equivalized income		0.015	0.073*	-0.003
		(0.032)	(0.036)	(0.036)
Financial literacy		-0.056	-0.122	-0.088
		(0.068)	(0.075)	(0.074)
Participant OPP		0.581***	-0.558***	0.309*
		(0.116)	(0.140)	(0.130)
Individual pension plan		-0.274*	0.089	0.430**
		(0.137)	(0.147)	(0.137)
Left-right ideology		0.127	0.241*	0.063
		(0.082)	(0.104)	(0.094)
Left-right ideology squared		-0.008	-0.015	0.001
		(0.008)	(0.010)	(0.009)
Trade union member		0.186	-0.287+	-0.015
		(0.129)	(0.170)	(0.151)
Risk aversion		-0.056**	-0.160***	-0.087***
		(0.022)	(0.025)	(0.024)
Austria (ref. Netherlands)		-2.780***	-1.311***	-1.162***
		(0.238)	(0.220)	(0.213)
Denmark		0.146	0.147	-0.142
		(0.182)	(0.247)	(0.243)
Germany		-2.537***	-1.015***	-1.129***
		(0.221)	(0.207)	(0.212)
Ireland		-0.000	0.023	0.029
		(0.159)	(0.209)	(0.207)
Spain		-1.771***	-1.046***	-0.813***
		(0.187)	(0.210)	(0.207)
Constant		-0.178	0.273	0.373
		(0.367)	(0.412)	(0.390)
Observations		3,325	3,325	3,325
Pseudo R^2	0.0745			

Note: Standard errors in parentheses; *** p<0.001, ** p<0.01, * p<0.05, + p<0.1

Figure A2. Linear models predicting the preferred proportion of public, occupational and private pension

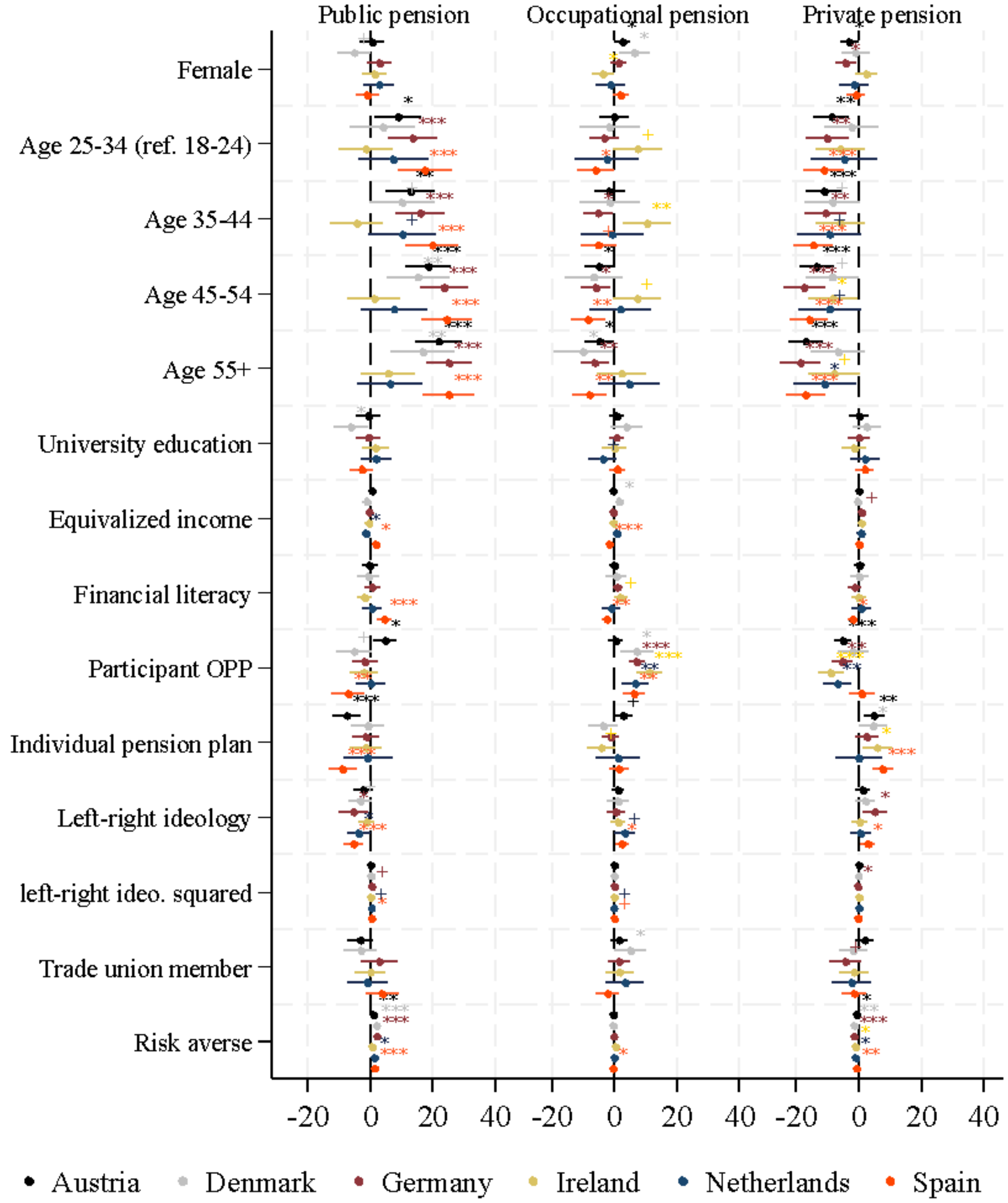

Note: + p < .1, * p < .05, ** p < .01, *** p < .001

Table A11. Percent imputed values by independent variable

Variable	Perc. imputed
Female	0.00
Age 18-24	0.00
Age 25-34	0.00
Age 35-44	0.00
Age 45-54	0.00
Age 55+	0.00
University education	3.19
Equivalized income	19.90
Financial literacy	4.42
Participant in OPP	9.57
Individual pension plan	0.00
Left-right ideology	9.63
Left-right ideology squared	9.63
Trade union membership	5.46
Risk aversion	5.30
